# Is Temperature Monitoring Necessary in Pediatric Circumcision?

**DOI:** 10.5152/eurasianjmed.2022.21071

**Published:** 2022-02-01

**Authors:** Cengiz Sahutoglu, Canan Bor, Zafer Dokumcu, Taner Balcioglu

**Affiliations:** 1 Department of Anesthesiology and Reanimation, Ege University School of Medicine, İzmir, Turkey; 2 Department of Pediatric Surgery, Ege University School of Medicine, İzmir, Turkey

**Keywords:** Heating systems, hypothermia, pediatric surgery, surgery duration

## Abstract

**Objective:** Perioperative hypothermia occurs in the pediatric age group in the perioperative period at a high rate. In this study, it is aimed to reveal the incidence of perioperative hypothermia and the risk factors that play a role in its development in pediatric circumcision cases that have a brief operation duration.

**Materials and Methods:** This prospective observational cohort study included 100 children who underwent circumcision under general anesthesia. All patients were heated with a passive heater and hypothermia was interpreted as a drop in body temperature below <36˚C. The patients were divided into 2 groups: group 1 (patients with body temperature <36˚C) and group 2 (≥36˚C). Demographic data, the American Society of Anesthesiologists’ Classification of Physical Health Score, premedication method, operation time, fluid amount, preoperative and postoperative temperature of patients as tympanic were recorded.

**Results:** The average age of the patients was 70 ± 40 months (median: 84) and 93% were ASA I. In 71% of patients, a decrease in body temperature and hypothermia developed in 39% compared to baseline. The duration of operation was longer in the group with hypothermia (30 min [15-70] vs. 25 min [15-60], *P* < .001). Only the duration of operation was determined as the independent risk factor associated with hypothermia (odds ratio: 1.103 [1.017-1.197], *P* = .018).

**Conclusion:** In this study, it was found that high rates of hypothermia developed even in minor operations such as pediatric circumcision. The risk of hypothermia increases with the prolongation of surgery.

## Main Points

- Hypothermia is defined as a decrease in a patient’s body core temperature below 36°C and is common during pediatric surgery.
- Children lose their body temperature faster than adults.
- Body core temperature of a patient is known to drop by 1-2°C following induction of anesthesia.
- Hypothermia can develop even in short-term surgeries.
- Hypothermia, even for a short time, can have negative outcomes in patients who have had minor or major surgery.

## Introduction

Intraoperative hypothermia is most commonly encountered in the pediatric age group. It has been shown that the body temperature lowers by 1-2°C in 1 hour following anesthesia induction.^1^ Compared to adults, pediatric patients’ core body heat lowers especially faster due to their high body-to-surface mass ratio. Heat loss occurs from either surface of the body or by airway due to radiation, conduction, convection, or evaporation. Hypothermia, independent of age, occurs in minor or major operations and can result in shivering, myocardial dysfunction, coagulopathy and increase in bleeding, wound infection, slower recovery from anesthesia, prolonged hospital stay, and higher hospital cost.^1-3^ Since hypothermia can develop even after operations with a short duration, it is important to monitor intraoperative heat in order to identify, prevent, and treat hypothermia in the early stages of surgery. Moreover, low operating room temperatures during surgery and anesthesia also cause intraoperative hypothermia.^2,3^

Even though active and passive heat management is conducted during surgery, intraoperative hypothermia incidence is still reported at a high rate (45-65%).^2-5^ Hypothermia incidence has been reported in 83.3% of newborns, 56.1% of babies, 46.4% of toddlers, and 54.4% of older children.^2^ Mild hypothermia (28-32°C) incidence is 1.2% and can cause serious complications especially during the newborn period.^2^ Patients’ age, body weight, duration of operation, the type of operation and anesthesia, intraoperative blood loss, and operating room temperature are all factors that contribute to intraoperative hypothermia.^2-4^ The primary objective of this study is to determine hypothermic incidence in a short operation such as circumcision. The secondary objective is determining the risk factors of hypothermia.

## Materials and Methods

After obtaining hospital ethical board approval from the Clinical Research Ethical Committee of Ege University School of Medicine (No: 15-6/11, Date: July 31, 2015) and consent forms from patients’ relatives, children under the age of 18 who were part of ASA I and II groups and who were circumcised under general anesthesia were included in the prospective, observational, and cohort research. Patients receiving other operations while under circumcision were excluded from the study. All patients’ electrocardiography, pulse oximetry, and non-invasive blood pressure were monitored. The body temperature of patients was monitored tympanically before and after the operation. Anesthesia induction was administered by sevoflurane (8%), O_2_, and air. After establishing vascular access, remifentanyl (0.5 mg kg^−1^), ketamine (0.5 mg kg^−1^), and dexamethasone (0.5 mg kg^−1^) were added. Maintaining anesthesia was ensured by sevoflurane (2-3%), extra dosage of remifentanyl (0.5 mg kg^−1^), or propofol (1 mg kg^−1^). Patients were administered age-appropriate standard of laryngeal masks (I-gel). After the area of operation of all patients was painted with povidone-iodine solution and covered in sterile green drapes, they were heated up with fan heaters (Bair Hugger, Arizant Health Care, Eden Prairie, Minn, USA). Before the circumcision incision, a penile block was placed with bupivacaine (dosage of 2 mg kg^−1^ on 0.25%). After the operation, all patients were given paracetamol 10 mg kg^−1^ as intravenous analgesic. Hypothermia was interpreted as body temperature dropping below <36 °C. Patients were split into 2 groups according to their postoperative temperatures: group I (patients with body temperature <36 °C) and group II (≥36 °C). Demographic data (age, weight, height, body surface area (BSA), body mass index (BMI) of patients), the American Society of Anesthesiologists’ classification of Physical Health score (ASA score), premedication method, operation duration, intraoperative liquid amount, preoperative (before induction) and postoperative temperature (in postoperative anesthesia recovery room) of patients were recorded tympanically. The primary objective of the study was set as determining the incidence of hypothermia, and the secondary objective was set as putting forward the risk factors pertaining to hypothermia.

Statistical Package for the Social Sciences for Windows 21.0 (IBM SPSS Corp.; Armonk, NY, USA) software was used for statistical analysis. Data were represented in terms of percentage (%, n), average ± standard deviation, or median (minimum–maximum). Kolmogorov-Smirnov test was used for the normal distribution of the data. In inter-group comparisons, chi-square and the Fisher exact test (gender, ASA score, and existence of premedication) were used for categorical variables. For quantitative variables, unpaired *t*-test (age and BSA) and Mann–Whitney *U* test (weight, height, BMI, duration of operation, amount of liquid used, and preoperative temperature) were used. Independent risk factors were identified by applying the binary logistic regression analysis (model: forward stepwise (conditional), model event: Omnibus test, *P* = .004, model fit: Hosmer and Lemeshow test, *P* = .140). Excluding multicollinearity (tolerance >0.2 and variance inflation factors <10), the age of the patient, BMI, the existence of premedication, preoperative temperature value, amount of liquid, and duration of operation were included in the model. Statistically, *P* < .05 was accepted as significant.

## Results

Among 100 patients included in the study, the average age was 70 ± 40 months (median: 84 months [2.5-156]). While 93% of the patients were ASA I, the remaining 7% were ASA II. In total, 75% of the patients were administered premedication (68% oral midazolam, 4% rectal midazolam, and 3% intravenous propofol). Baseline body temperature decrease was observed in 71% of the patients (36.4 ± 0.4°C vs. 36 ± 0.45°C, *P* < .001). Hypothermia (<36°C) developed in 39% of all patients. One of the patients’ temperature value was <35°C (34.4°C) (Table 1).

**Figure 1.**
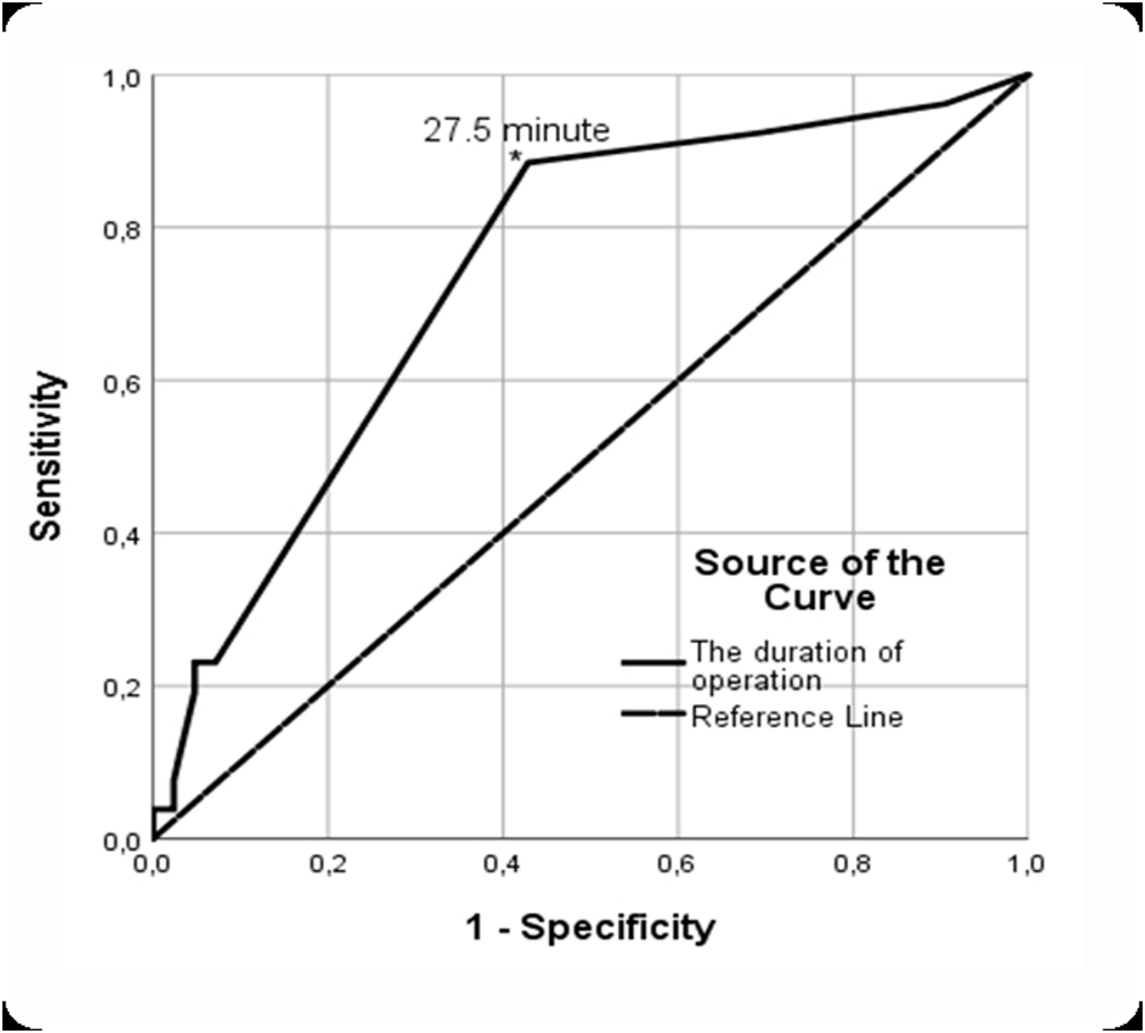
Receiver operating characteristic curve of the hypothermia.

In the hypothermia group, age, weight, height, BMI, BSA, and preoperative temperature values were lower while premedication rate and the amount of liquid used were higher. However, these parameters were not found to have any statistical significance (*P* > .05). The average duration of operation was 29 ± 9 minutes (between 15 and 70) and was longer in the hypothermia group (30 minutes [15-70] vs. 25 minutes [15-60], *P* < .001). In the logistic regression analysis, only operation duration (odds ratio (OR): 1.103 [1.017-1.197], *P* = .018) was found statistically significant as an independent risk factor for hypothermia (Table 2). The cut-off value for hypothermia was determined as 27.5 minutes (area under the curve (AUC): 0.741, *P* < .001, 95% CI: 0.626-0.857). However, the sensitivity of duration was at 89.7%, while specificity remained at 55.6% (Figure 1).

## Discussion

This study has determined that hypothermia can develop at a high rate (39%) even in short operation durations and an operation that lasts 30 minutes or longer is sufficient for hypothermia development.

Although passive and active heating were administered during operations, Lai et al^2^ found intraoperative hypothermia incidence as 46.6%. The patient’s age and weight, operation duration, operation scale, intraoperative blood loss, form of anesthesia, and operating room temperature were determined to be contributing factors to hypothermia. Hypothermia occurred more commonly especially in major surgeries with longer duration. In order to keep the body temperature of the patient at the normothermia level, using various active and passive heating methods prior to, during, and after all operations (including minor operations) was recommended. Pearce et al^4^ have determined the incidence of hypothermia in the pediatric age group as 52%. Invasive procedures, older age, long duration of anesthesia, large amount of blood loss, and blood transfusion were determined to be risk factors for hypothermia.

Yang et al^6^ found 25.7% of hypothermia prevalence in all age groups (male 27.1% and female 23.8%, *P* = .100). According to multivariate logistic regression analysis, increased age and operation rooms with laminar flow pose a risk for hypothermia while operations except general surgical operations were found to be less risky. Compared to traditional operating rooms, hypothermia developed more frequently at 1.53 times (95% CI: 1.19-1.96, *P* = .001) in operating rooms with laminar air flow and 2.58 times (95% CI: 1.64-4.06, *P* < .001) in patients over the age of 60. Hypothermia prevalence was found as 17%, 13.7%, and 22.3%, respectively, in 30, 60, and 90 minutes post-induction. In surgeries lasting less than 2 hours, between 2 and 3 hours, and more than 3 hours, the prevalence of hypothermia was found as 14.9%, 21.1%, and 29.6%, respectively. Most hypothermia cases were observed in general surgical operations (37%) and urological operations (30.4%).

The patients included in our study underwent circumcision surgery, and the maximum operation time in the hypothermia group was 70 minutes (median 30 minutes). A moderate decrease was observed in the basal temperature of 71% of our patients, while the hypothermia incidence (<36°C) was determined as 39%. This rate is high for an operation with a short duration such as circumcision. This high rate is traced to active–passive heating systems not being used effectively. The reason for this was attributed to the forced-air pre-warming body not covering under the umbilicus. Because the surgical procedure was simple and short, the surgery did not involve body cavities, did not require blood transfusion, and the fluid requirement was low, the incidence of hypothermia did not increase. We determined that hypothermia can develop even in simple and short operations like circumcision and that this situation has been overlooked. It is found that the duration of the operation being 27.5 minutes and longer poses a risk for hypothermia and the incidence increases as this duration increases. No correlation between basal temperature and hypothermia was found.

Morehouse et al^5^ reported that 40% (n = 43) of the 108 babies who were admitted to the neonatal intensive care unit (NICU) developed hypothermia after the operation; 51% of the patients were operated in the operating room, while 49.1% were operated in the NICU. The perioperative hypothermia rate was found to be 7 times higher in the operating room group compared to the NICU (65.5% vs. 13.2%, *P* = .008). Similarly, the probability of hypothermia development in the intraoperative and postoperative period in the OR group was determined to be 10 times higher compared to the NICU group (*P* = .001). The hypothermic group developed 6 times more inspiratory complications (*P* = .025), and these patients were reported to need thermoregulation, cardiac, and inspiratory intervention (*P* < .05).

Kim et al^7^ reported the hypothermia incidence as 8.9% in their quality development study including 7532 patients observed over a period of 66 weeks. They identified that 78% of the patients who developed hypothermia were at 35.50-35.99°C and were in the 12-18 age group. Hypothermia was observed most commonly in orthopedic surgery at 46% and was observed in pediatric surgery at 26%. After using interventional operations (room temperature being above 23.9°C, using fanned active heating systems, radiant heaters, heating pads, and moisturizers etc.), hypothermia incidence was lowered to 4.2%.

Schroeck et al^8^ reported in studies in 2006-2014 including 2350 patients that 82% of the patients were normothermic, 9% were hypothermic (<36°C), and 9% were hyperthermic (>37.5°C). Only 1.1% (26 patients) were found to be <35°C. Comparing the pre-2010 data when no active heating was administered with the post-2010 data when active heating was administered, hypothermia incidence dropped from 24% to 2% (*P* < .0001), while hyperthermia incidence was statistically insignificant (from 13% to 8%, *P* = .357). Hypothermia was found to be higher in high ASA scores (OR 1.7, 95%: 1.1-2.6; *P* = .02).

Tander et al^3^ have stated that in all patients, there is a significant decrease in central core temperature 10 minutes after anesthesia induction in comparison to initial temperature. The largest decrease of temperature in newborns and infants was observed in major operations and low operating room temperatures (<23°C). It was determined that major operations factored 2.66 times and operation room temperature being <23 °C factored 1.96 times in the risk of hypothermia.

Unlike other studies, factors except for the duration of the operation did not contribute to hypothermia. Operating rooms in this study were kept at 21-24°C. Heating via active fan heating systems was started after the patients were covered with a sterile sheet and continued until the end of the operation. However, only 1 patient had a temperature below 35°C and no early postoperative complications were encountered. Temperature measurements of all patients were repeated in the postoperative period, and patients who developed hypothermia continued to be heated actively (active fan heating systems) and passively (blanket) in the postoperative period. All patients were taken to service follow-up from postanesthesia care unit as normothermic.

In addition to perioperative warming, preoperative warming can be an important adjunct measure in the prevention of hypothermia. It has been reported that active or passive warming applied 30-60 minutes before the preoperative period was associated with a significant decrease in perioperative hypothermia.^9^ De Witte et al^10^ examined the patients in 3 groups as forced-air prewarming, carbon fiber resistive heating, or no prewarming and found that there was no difference between the intervention groups and the control group. However, in this study, forced-air prewarming did not include shoulders, feet, or ankles. Therefore, it caused hot air to escape and caused insufficient heating. John et al^11^ compared resistive heating and forced-air warming and reported that 54% of the patients developed hypothermia in the resistive heating group and 36% in the forced-air warming group (*P* = .017). The temperature was lower in the resistive heating group (35.9°C vs. 36.1°C, *P* = .029). The authors stated that forced air-warming was more effective than resistive heating in preventing hypothermia.

The patient being uncovered to establish peripheral vascular access, vasodilatation caused by anesthetic medicine, cold intravenous liquid used in transfusion, and liquids used for sterilization are especially thought to be contributing factors in the development of hypothermia. Pre-induction warming or warming of fluids was not considered in our patients due to the short operation time. Instead, it used passive heating (covering the body in sheets) and active heating (fan hot air heating systems) in order to prevent hypothermia. Active heating systems were not sufficient because they did not cover the whole body.

This study has several limitations: First, the study was designed as a prospective, observational study and it aimed to detect the incidence of perioperative hypothermia within the routine study. Second, active and passive heating could only be used after surgical sheets and body temperature of the patients was not constantly monitored, hypothermia was observed at the end of the operation when measured tympanically. Third, complications related to hypothermia were not defined. Finally, due to the circumcision operation, it was performed only in the male gender. Therefore, gender comparison could not be made.

In conclusion, perioperative hypothermia incidence is not low in pediatric patients. Hypothermia incidence increases in prolonged operation duration. It should be kept in mind that hypothermia can develop even in simple and short operations such as circumcision, and all heating systems, passive and active, should be used in order to protect the patients from hypothermia.

## Figures and Tables

**Table 1.** Demographics and Intraoperative Values of the Patients

	Group I (Hypothermia, n = 39)	Group II (Normothermia, n = 61)	*P*
Age (months)	63.5 ± 36.2	74.5 ± 42	.179
Weight (kg)	17.5 (9-54)	24 (3.5-101)	.119
Height (cm)	114 ± 21	119 ± 26	.334
BSA (m^2^)	0.84 ± 0.25	0.93 ± 0.36	.117
BMI (kg m−2)	16 (10-21)	16 (10-33)	.395
ASA score (I/II)	38/1	55/6	.242
Premedication (yes)	84.6 (%)	68.9 (%)	.076
Duration of operation (min)	30 (15-70)	25 (15-60)	<.001*
Fluid amount (mL m−2 h−1)	350 (114-860)	329 (90-930)	.950
Preoperative temperature (°C)	36.3 (35.8-37.3)	36.4 (36-37.8)	.536

BSA, body surface area; BMI, body mass index; ASA, American Society of Anesthesiologists. *: p<0.05. Results were presented as mean ± SD, n, %, and median (min-max).

**Table 2.** Independent Risk Factors for Hypothermia (Binary Logistic Regression Analysis)

	*B*	SE	Wald	*P*	OR	95% CI
Duration of operation (min)	0.098	0.042	5.614	.018*	1.103	1.017-1.197
Constant	−3.301	1.215	7.382	.007*	0.037	

*B*, coefficient value; SE, standard error of coefficient; OR, odds ratio. *: p<0.05.
